# Allen's Silicious Cement

**Published:** 1852-04

**Authors:** 


*Allen's Silicious Cement.*—We have received from Professor Allen, of Cincinnati, Ohio, a set of artificial teeth, designed for the upper jaw, mounted very beautifully on a platina base; to which, in the interdental spaces, and on the buccal and palatine surfaces of the teeth, a siliceous composition is fused, giving to the piece a very finely finished appearance. The teeth were first attached to the plate in the usual way, and the silicious composition afterwards applied and fused in a furnace. The teeth being sustained by backings soldered to the plate, their liability to be broken from the base is, certainly, very greatly diminished, and when mounted in this manner, they unquestionably possess, on some accounts, advantages over those put up in the ordinary way. The complete exclusion of the secretions of the mouth from between the teeth and plate, by the intimate union of the two, must prevent the contamination of the breath which so frequently arises from the use of artificial teeth, and especially when the most constant attention is not paid to their cleanliness.

But notwithstanding these apparent advantages, it is supposed by many, that the springing of the plate during mastication, will crack the cement, and, in a short time, cause it to scale off, but the result of the experiments which have been made with it thus far, so far as it has come to our knowledge, would seem to prove this opinion to be incorrect. But this is a point which time will soon settle, and until then, it is useless to speculate upon the subject. Admitting this supposition to be groundless, as we sincerely hope it will prove to be, the introduction of this method of mounting artificial teeth in the United States, will prove a new epoch in mechanical dentistry in this country, which may be truly said to have ***progressed***, during the last twenty-years, ***a pas de geant***. We cannot, however, but regard the use of platina as a base for artificial teeth as objectionable, the weight of it being about double that of gold.

